# Nutritional Importance of Selected Fresh Fishes, Shrimps and Mollusks to Meet Compliance with Nutritional Guidelines of n-3 LC-PUFA Intake in Spain

**DOI:** 10.3390/nu13020465

**Published:** 2021-01-30

**Authors:** Maria Dolores Mesa, Fernando Gil, Pablo Olmedo, Angel Gil

**Affiliations:** 1 Biomedical Research Center, Department of Biochemistry and Molecular Biology II, Institute of Nutrition and Food Technology “José Mataix”, University of Granada, Parque Tecnológico de la Salud, Avenida del Conocimiento s/n, 18100 Armilla, Spain; agil@ugr.es; 2 Biosanitary Research Institute of Granada, Ibs, 18012 Granada, Spain; 3 Department of Legal Medicine and Toxicology, School of Medicine, University of Granada, Avda. de la Investigación 11, 18016 Granada, Spain; fgil@ugr.es (F.G.); polmedopalma@ugr.es (P.O.); 4 Spanish Biomedical Research Networking Centre, Physiopathology of Obesity and Nutrition (CIBEROBN), Instituto de Salud Carlos III, Monforte de Lemos 3-5, 28029 Madrid, Spain

**Keywords:** fishery products, omega 3, fatty acids, docosahexaenoic acid, eicosapentaenoic acid

## Abstract

Fishery products are the main source of dietary n-3 long-chain polyunsaturated fatty acids (n-3 LC-PUFA). Following the European Commission’s request to address the risks and benefits of seafood consumption, and taking into account the great variability of nutrient and contaminant levels in fishery products, the present work aims to estimate the n-3 LC-PUFA provided per serving of selected fishes, shrimps and mollusks that are commonly consumed in Spain. This would enable the establishment of a risk–benefit analysis of fish consumption and provide recommendations for fish intake to comply with nutritional guidelines of n-3 LC-PUFA intake. We confirmed high variation in the pattern and contents of fatty acids for different species. n-6 PUFA were minor fatty acids, whereas palmitic (C16:0), oleic (C18:1 n-9), and mainly eicosapentaenoic (C20:5 n-3) and docosahexaenoic (C22:6 n-3) acids were the major fatty acids in the sample. Therefore, consumption of 2–3 servings per week of a variety of fishery products may contribute to compliance with the recommended daily n-3 LC-PUFA intake while maintaining an adequate balance to avoid contaminant-derived potential risks (metals and others). Taking the fatty acid content of fishery products described in this study into consideration, it is advisable to include one serving of fatty fish per week in order to meet recommended n-3 LC-PUFA levels.

## 1. Introduction

The nutritional importance of n-3 long-chain polyunsaturated fatty acids (n-3 LC-PUFA), also known as omega-3 fatty acids, and particularly eicosapentaenoic acid (EPA, 20:5 n-3) and docosahexaenoic acid (DHA, 22:6 n-3), derives from their metabolic implications in different physiological and pathological mechanisms [1]. n-3 LC-PUFA are present in membranes and influence their structure and functions. They are key nutrients for growth and maturation during pregnancy and early life [2]. Furthermore, they are precursors of many anti-inflammatory mediators, i.e., eicosanoids and docosanoids, and they also modulate the expression of key genes [3]. Thus, n-3 LC-PUFA may contribute to the prevention and treatment of a number of inflammatory-based non-communicable chronic diseases such as cardiovascular disease, cancer, lung disease, and inflammatory bowel disease [4,5,6]. In addition, n-3 LC-PUFA may contribute to the reduction in plasma triacylglycerols, decrease blood pressure, and contribute to the regulation of thrombogenesis [1,7]. Although EPA and DHA are not essential fatty acids, their intake is considered to be essential under certain physiological and pathological circumstances [8]. Based on this scientific evidence, the European Food Safety Authority (EFSA) Panel on Dietetic Products, Nutrition and Allergies (NDA) approved several health claims in relation to the cardioprotective effect of n-3 LC-PUFA and their beneficial effects on the brain and eyes [9,10].

Fishery products are the main dietary source of EPA and DHA. Their composition depends on the fish species, the geographical origin, and even the season of fishing [11]. Indeed, the NDA EFSA Panel concluded that consumption of 1–2 servings of seafood a week, and up to 3–4 servings a week during pregnancy, is associated with better functional outcomes of neurodevelopment in children, and with a lower risk of coronary heart disease mortality in adults [12]. However, although fish is a good source of many nutrients, it also may contain significant amounts of some environmental contaminants, especially metallic elements such as methylmercury. In previous studies, we reported the content of metallic, metalloids and essential minerals in fishery products marketed in Spain [13,14]. Due to the variability of nutrient and contaminant levels in seafood, it is recommended that each country establishes its own pattern of fish consumption, especially considering the species of fish consumed, and carefully assesses the risk of exceeding the tolerable intake of methylmercury while obtaining the health benefits from consumption of seafood, mainly n-3 polyunsaturated fatty acids. Here, we carry out this assessment for Spain for the first time, which is the novelty of the present work. Limiting consumption of fish species with high methylmercury content is the most effective way to achieve the health benefits of fish whilst minimizing the risks posed by excessive exposure to methylmercury [15]. Thus, the aim of the present study was to estimate the n-3 LC-PUFA provided per serving of selected fish, shrimps and mollusk species with different origins in order to clarify whether the benefits of the intake of fishery products exceeded the potential risks as recommended by EFSA, and to suggest an appropriate intake of n-3 LC-PUFA to comply with international nutritional guidelines.

## 2. Materials and Methods 

### 2.1. Reagents and Materials 

A fatty-acid standards mix (47885-U from Supelco) and acetyl chloride were purchased from Sigma-Aldrich (Química S.A., Spain). Hexane, methanol, benzene, potassium and carbonate were acquired from Panreac Química (Darmstadt, Germany). 

### 2.2. Samples

A total of 275 samples from whole fresh fishes, shrimps and mollusks (*n* = 11 per species) were collected for the present study, with species included according to their frequent consumption in Spain [16]. Table 1 details the species included, their origin, the season of fishing, and the amount of fat they contain, as described in the Spanish Food Composition Databases (BEDCA) [17], although, for the blue shark, this was from the United States Department of Agriculture Food Composition Databases (USDA) [18]. We considered these values for the estimations included in the present work. Fishery products were purchased in the central market of Granada in southern Spain. The origin of fresh species was 29.1% from Andalusia, 40.0% from other places in Spain, including the Mediterranean Sea and Atlantic Ocean, 9.5% from other places in Europe, and 21.4% from the rest of the world. The exclusion criteria were: 1) uneven size or length with respect to the species average and 2) non-fresh samples. In addition, frozen codfish from the Norwest Atlantic Ocean were included because they are commonly consumed in the inland areas of Spain. Immediately after purchasing, sampling was performed following Commission Regulations (EC) No 1882/2006 amended by EC No. 629/2008 and EC No. 420/2011. Generally, muscle meat of fish was used. In crustaceans, muscle meat from appendages and the abdomen were sampled, and in the case of crabs and crab-like crustaceans, muscle meat from appendages was sampled. Cephalopods were analyzed without viscera. All samples were stored immediately in a freezer at −32 °C until samples were collected, and then at −80 °C until analysis. 

### 2.3. Lipid Isolation and Fatty Acid Methylation

We mixed 100 mg of lyophilized sample with 4.5 mL of water and 0.5 mL of MgCl_2_ (0.25%, *w*/*v*), and extracted lipids by adding 3 mL of isopropanol containing 50 mg/L of BHT and 2 mL of hexane in continuous agitation for 2 min. Samples were centrifuged at 3500× *g* for 10 min at 4 °C. Then, the organic layer was re-extracted three times with 2 mL of hexane. The hexane extract was evaporated to dryness under nitrogen. The isolated lipids were dissolved into 2 mL of methanol/benzene (4:1 *v*/*v*). Methylation was carried out as previously described [19]. Briefly, samples were added to 200 µL acetyl chloride and heated at 100 °C for 1 h. After cooling, 5 mL of 0.43 M K_2_CO_3_ was added to stop the reaction and neutralize the mixture. Tubes were then shaken and centrifuged, and the benzene upper phase was dried under nitrogen and resuspended with 100 µL hexane.

### 2.4. Quantification of Fatty Acids and Data Calculations

We injected 1 µL of the methylated fatty acids in an Agilent A7890 gas–liquid chromatographer (Agilent Technologies, Palo Alto, CA, USA) coupled to mass spectrometry detection (Masas Quattro GC from Waters, Milford, MI, USA) (GS-MS) with a capillary column SP 2560 (100 m × 0.25 mm inner diameter; 0.20 µm film thickness) (Supelco, Bellefonte, CA, USA) to analyze the fatty acid composition [20]. Fatty acids were identified by comparing them with predetermined retention times of injected individual fatty acid standards. Retention times are specified in Supplementary Table S1. The fatty acid profile for each species was expressed as the mean percentage ± SEM. Data are presented in percentages as the mean ± SEM. 

To calculate the amount of total fatty acids per serving (g fatty acids/150 g fish) allowance must be made for the fact that the total fat in a food includes triglycerides (of which a proportion is glycerol, i.e., not fatty acid), phospholipids and unsaponifiable components such as sterols. In foods where the total fat is virtually all triglyceride, a correction factor based on the average chain length of the fatty acids present is adequate [21]. Therefore, to calculate the amount of total fatty acids per serving (g fat/150 g), we multiplied the amount of total fat described in BEDCA [17] and USDA (for the blue shark) [18] by the suggested correction factor: 0.9 for fatty fish and medium fatty fish, and 0.7 for lean fish, and mollusks and crustaceans, which have been considered as lean fish.

To convert percentages of individual fatty acids to mg/g fish (included in Supplementary Tables S2–S4), we multiplied the percentage of each individual fatty acid by the amount of total fatty acids calculated for the fish and divided by 100. Individual n-3 LC-PUFA were added to calculate total n-3 LC-PUFA (mg/g fish).

To calculate the amount of n-3 LC-PUFA (g) per serving of seafood (150 g) we multiplied the amount of n-3 LC-PUFA fatty acids per g fish by 150 g.

To calculate the mg/d of n-3 LC-PUFA provided by the ingestion of one serving per week we divided the amount of total n3- LC-PUFA provided by one serving per 7 days and expressed in mg/d.

## 3. Results

Fishes were classified into fatty (6–25% fat), medium fatty (2.5–6% fat) or lean (<2.5% fat) depending on their lipid content according to BEDCA and USDA data (Table 1). Table 2 describes the percentage of individual saturated fatty acids (SFA), monounsaturated fatty acids (MUFA), n-6 PUFA and n-3 PUFA in selected fatty and medium fatty fishes. Table 3 reports the percentages of individual SFA, MUFA, n-6 PUFA and n-3 PUFA in selected shrimps and mollusks.

Among fatty fishes, mackerel, sardine and anchovy had more SFA (>40%), which was mainly palmitic acid (C16:0), whereas salmon and gilt-head had more MUFA (67% and 35%, respectively), which was mostly oleic acid (C18:1 n-9). n-6 PUFA represented a minor proportion of the fatty acids in all fishes (Table 2), mainly linoleic acid (C18:2 n–6) in salmon and sardine and arachidonic acid (C20:4 n–6) in mackerel, gilt-head, and anchovy. The amount of EPA ranged from 1.9% in salmon to 7.7% in anchovy, while the amount of DHA ranged from 3.0% in salmon to 22.8% in anchovy (Table 2). 

Among the medium fatty fishes, blue shark, red mullet, and tuna had more SFA (35–49%), which was mainly palmitic acid (C16:0), whereas swordfish had more MUFA (67%), which was primarily oleic acid (C18:1 n–9). N–6 PUFA were minor fatty acids in all fishes, and mainly comprised arachidonic acid (C20:4 n-6) (Table 2). The amount of EPA ranged from 0.9% in swordfish to 10.2% in red mullet, and the amount of DHA ranged from 9.2% in swordfish to 21.3% in blue shark (Table 2).

Among the lean fishes (Table 3), scad (horse mackerel), megrim, perch, sole and pangasius had more SFA (37–45%), which was mostly palmitic acid (C16:0); hake and European seabass had more MUFA (44% and 42%, respectively), mainly oleic acid (C18:1 n-9); and codfish, European hake, blue whiting and anglerfish had more n-3 PUFA (52%, 44%, 44% and 36%, respectively), which was mainly DHA. n-6 PUFA were minor fatty acids in all fishes. Arachidonic acid (C20:4 n-6) was the major n-6 PUFA in these fishes, except for scad, European seabass, and pangasius. The amount of EPA ranged from 0.2% in pangasius to 10% in blue whiting, and DHA ranged from 1.3% in pangasius to 35% in European hake (Table 3). 

Among the shrimps and mollusks (Table 4), mussels and clams had more SFA (41% and 48%, respectively), mainly palmitic acid (C16:0), while shrimps, squid and cuttlefish mostly contained n-3 PUFA (36%, 54 % and 43%, respectively), which was mainly DHA. n-6 PUFA were minor fatty acids in all analyzed seafood samples, mainly arachidonic acid (C20:4 n-6), which ranged from 0.7% in squid to 5.1% in shrimps. DHA was the main n-3 PUFA in all samples (ranging from 17% in clams to 42% in squid except mussels, which contained a higher percentage of EPA (19%) than DHA (Table 4).

Table 5 shows the estimated amount of n-3 LC-PUFA provided per gram of seafood and per serving (considered as 150 g of fishery product [21]). In addition, we estimated the amount of n-3 LC-PUFA (mg/d) provided after the ingestion of one serving per week of fatty, medium fatty, and white fishes, mollusks, and crustaceans. Fatty fishes are the main source of n-3 LC-PUFA per gram or serving, with the most provided by mackerel, anchovy, sardine, gilt-head, and salmon. Blue-shark and red mullet also provide similar amounts to sardine and salmon. With regard to fresh mollusks and crustaceans, squid provides the highest amount of n-3 LC-PUFA (Table 5).

## 4. Discussion

Fishes, crustaceans and mollusks are a good source of proteins and essential minerals. However, individuals who eat no fish will have most difficulty in meeting the daily recommended intake of n-3 LC-PUFA, which may affect cardiovascular health and fetal development [8]. However, these animals may contain methylmercury, dioxins, and other xenobiotics as contaminants, which represent a health risk [1,14]. The present work aimed to estimate the n-3 LC-PUFA provided per gram and serving of selected fish, shrimps, and mollusk species to provide approximated data on how each species contributes to meeting the recommended intake of n-3 LC-PUFA to comply with international nutritional guidelines. 

The essential nature of fatty acids was discovered more than 80 years ago. The essential fatty acids (EFAs) for humans are linoleic acid (18:2 n-6) and α-linolenic acid (18:3 n-3), while arachidonic (20:4 n-6) and DHA fatty acids, which are synthesized from them, are considered “conditionally” essential [23,24,25]. These fatty acids must be present in the diet in quantities and proportions that are relatively well established. EFAs and their LC-PUFAs derivatives are important components of human cell membranes and precursors of bioactive molecules, thus explaining their importance. The effects of EFAs and LC-PUFAs are mediated not only by their known impacts on membrane biophysical properties and the corresponding electrophysiological correlates, but also by effects on cell growth, differentiation, and functional maturation, and by modulating gene expression during development and at all subsequent stages of human life [1,26,27].

The n-6 and n-3 fatty acids, particularly those of greater length and unsaturation, such as arachidonic acid and DHA, are required for the development and functionality of the nervous and visual systems [28,29]. Similarly, some derivatives of LC-PUFA, namely prostacyclins, thromboxanes, leukotrienes, and other oxidized compounds, are involved in the control of vascular homeostasis, with the eicosanoids derived from n-6 and n-3 fatty acids generally acting antagonistically. Resolvins, which are derived from n-3 fatty acids, are also involved in the resolution of the inflammation process [30,31]. The active participation of EFAs and LC-PUFAs in the regulation of the expression of different genes has also been reported. In particular, by acting as ligands for several transcription factors, they are implicated in causing a high prevalence of non-communicable chronic diseases such as obesity, type 2 diabetes and dyslipidemias, as well as other conditions of a genetic origin [1,32].

The time and site of the catch also influence fishery products’ fatty acid profiles [33,34]. This is related to different factors, including the temperature and salinity of the water, the fatty acid composition of the available diet, and the biological cycle [33]. The samples we analyzed were caught in different seasons of one year. In addition, although the pieces were purchased in the central market of Granada (southern Spain), their origin varied according to their availability in the market (Table 1). Overall, our results confirmed that, among fish, (1) there is great variation in fatty acids across species, so their contribution to recommended intakes should be accounted for by dietary recommendations; (2) n-6 PUFA are minor fatty acids in the overall composition; (3) palmitic acid is the major SFA; (4) oleic acid is the major MUFA; (5) DHA is the major n-3 PUFA. Therefore, the intake of these fishery products may help to enhance the intake of n-3 LC-PUFA and provide the associated health benefits. 

Previous studies have reported that the fatty acid composition of Atlantic salmon tissues [35], gilt-head [36], and sardine [33] reflect the fatty acid content of their feed. In addition, it has been reported that the fatty acid composition of sardines [33], mackerel [37], swordfish [38] and scad [39] varies significantly with season and catch site. Our results agree with previous fatty acid profiles described for red mullet [40], tuna [41], megrim [42], European seabass [43], common sole, European hake [44] and blue whiting [41]. However, other authors have reported different fatty acid profiles for sardine [33,45], gilt-head [36], anchovy [41,46], anglerfish [40], and European hake [41]. In some of these studies, the authors did not indicate the origin of the samples or the season of catch, which could partly explain the differences with the present study. Thus, it is worth reemphasizing the importance of describing the origin and time of catch when describing the fatty acid profile of fishery products. 

In accordance with our results, when analyzing freshwater fish, different fatty acid profiles have been described for perch [47]. Although perch is a freshwater fish, it contains a considerable amount of n-3 PUFA, in contrast to pangasius, another freshwater fish that provides very little of this fatty acid. This may reflect feeding differences between the species. Young perch feed on plankton, while adults are carnivorous. In contrast, pangasius feed on the remains of dead fish, powdered bones, and vegetal flour. Thus, the fatty acid profile of pangasius is characterized by very low levels of n-3 PUFA. However, it has been demonstrated that including 100% linseed oil in the diet of juvenile pangasius may increase the presence of n-3 PUFA in muscle tissue [48]. From this point of view, the fatty acid composition depends on the fishery diet. Therefore, this should be considered when feeding farmed fish in order to improve their fatty acids profile and reduce the presence of contaminants.

Shrimps and bivalves provide amounts of n-3 LC-PUFA similar to those of many fishes, while mollusks are a better source of these fatty acids. However, these kinds of seafood provide less fat, and, consequently, n-3 LC-PUFA, than fatty and medium fatty fishes. As reported previously, palmitic acid and DHA are the major fatty acids present in shrimps, although the composition varies across the different seasons of the year [34]. The shrimps analyzed in our study were caught in the Atlantic coast of Andalusia, whereas samples analyzed by Ayas et al. [34] were from the Mediterranean Sea in the same season. This might explain the higher amount of n-3 PUFA found in our shrimps, since zooplankton production in the Mediterranean Sea has been described as poor compared to that in the Atlantic zone [33]. A low amount of MUFA in relation to SFA and n-3 PUFA has previously been reported in mussels [49] and clams [50]. Fokina et al. [51] described a “homeostatic adaptation” to maintain the membranes in response to temperature fluctuations in mussels. On the other hand, a similar fatty acid profile, characterized by high levels of both EPA and DHA, has been observed in squid in the eastern Pacific Ocean [52] and cuttlefish caught off the coast of the Canary Islands [53]. 

Although fish and shellfish are the major contributors of methyl mercury exposure in the diet, our research group has reported that, in general, the consumption of these products can be regarded safe, although regular or excessive consumption of predatory fish species may involve a risk [14]. The highest mercury concentrations were found in predatory species, namely, catshark, swordfish, tuna, and blue shark. More than 99.9% of the total mercury found in these species was in the form of methyl mercury (MeHg). Other species with exceptional levels were anglerfish, perch, and scad. However, mercury concentrations were generally below the maximum level (ML) set by the European Community (EC) regulations for each metal in different foodstuffs, with only 1.24% of samples belonging to the predatory fish species exceeding that level. Regarding cadmium, bivalve mollusks such as mussels and clams presented the highest levels for fresh products. The species with the lowest Cd concentrations were European hake, salmon, red mullet, megrim, and blue whiting. Cadmium concentrations were below the ML in almost all cases, with only 0.62% exceeding the limit. Foodstuff categories with higher ML, such as bivalve mollusks and cephalopods, presented the highest cadmium concentrations. More than 70% of the samples analyzed showed lead levels below the limit of detection (LOD). The only fresh products showing levels above LOD were cuttlefish, European hake, and common sole. No fish species showed median lead levels that exceeded the ML set by the EC for fish and crustacean samples. No ML is currently set by the EC for arsenic. The fresh products with higher arsenic levels were shrimp, sardine, and red mullet. Other species worth noting were catshark, clam, scad, common sole and mussel [14]. Therefore, according to the sum of their median levels of mercury, cadmium, lead, and arsenic, the species with the highest toxic metal content was catshark, while pangasius and codfish had the lowest metal concentrations [14]. Indeed, recommended fish and shellfish intake does not pose a health risk to consumers [14], and these fishery products are a reasonable source of essential metals such as copper, manganese, zinc, and, particularly, selenium [13]. Indeed, fishery products provide a healthy mercury:selenium balance, except for shark species and other predatory species, which make them less suitable for consumption than other species with a strong content in essential elements, such as mussels, scad, sardine, and anchovy [13], especially in pregnant and lactating women and during early life [15]. Thus, taking into consideration the fatty acid content and nutritional value of the fishery products analyzed in the present study, together with the median sum of essential and toxic elements described [13,14], the most beneficial species for general consumption are fatty fish such as mackerel, anchovy, gilt-head, sardine and salmon, which provide an appropriate mercury:selenium balance while offering high content of n-3 LC-PUFA. Regarding mollusks and crustaceans, squid is the best source of n-3 LC-PUFA.

The EFSA Panel established that, when consumed as part of a balanced diet, 250 mg/d of EPA plus DHA can be recommended to maintain normal brain function and vision [9,10], while 250–566 mg/d would cause a reduction (36–37%) in the risk of CHD mortality [12]. Indeed, to provide this amount of n-3 LC-PUFA, the EFSA recommends the consumption of about 1–2 servings of fishery products per week to reach nutritional requirements, and no more than 3–4 servings per week to avoid adverse effects derived from the accumulation of contaminants found in fishery products [14]. Our data show that, in general, 1–2 servings of fatty fish and some mollusks and crustaceans would reach the dietary n-3 LC-PUFA recommendation, but at least three servings of lean fish and mollusks and crustaceans would be needed. Therefore, with the purpose of better adjusting nutritional recommendations, and considering that fishery products are a nutritional source of n-3 LC-PUFA, it is worth considering the possibility of revising and adjusting recommendations for some individual kinds of seafood, taking into account the contaminants and n-3 LC-PUFA levels some species contain. Our results show that the consumption of 2–3 servings per week of a variety of fishery products may contribute to reaching the recommended daily n-3 LC-PUFA intake. Thus, considering the fatty acid content of the fishery products described, it is advisable to include one serving of fatty fish per week to guarantee n-3 LC-PUFA recommendations are met.

One limitation of the present stud is that we did not determined fat fish contents and our calculations are based on previously published data [17,18,21]. Therefore, and due to the great variability of fish composition, our data are approximate estimations and must be considered as a guide.

## 5. Conclusions

Our results confirm the high levels of variation in fatty acid content of different species of fishery products that provide a substantial amount of n-3 LC-PUFA, mainly DHA. Due to this variation, consumption of 2–3 servings per week of a variety of fishery products may help meet the recommended daily intake of n-3 LC-PUFA in adults, including in pregnant and lactating women. This would help maintain an adequate balance to avoid potential risks caused by environmental contaminants present in these species. It is advisable to revise and slightly adjust some individual fishery product recommendations, including at least one serving of fatty fish per week, to guarantee n-3 LC-PUFA recommended intake levels are met.

## Figures and Tables

**Table 1:** Origin and fat composition of fatty, medium fatty, and white fresh fishes, mollusks and crustaceans usually consumed in Spain.

	Scientific Name	Origin	Season of Fishing	Fat Composition (g/100 g)
Fatty fishes				
Salmon	*Salmo salar*	Norway (*n* = 11)	WI; SP; SU	12.0
Mackerel	*Scombers combrus*	Spain: Andalusia (Med. Coast (*n* = 8), Atl. Coast (*n* = 1), Basque Country (*n* = 2)	WI	11.9
Sardine	*Sardina pilchardus*	Spain: Málaga (*n* = 11), Huelva (*n* = 1)	WI	7.5
Gilt-head	*Sparus aurata*	Spain: Alicante (*n* = 4), Murcia (*n* = 4), Huelva (*n* = 3)	WI; SP; SU; AU	7.2
Anchovy	*Engraulisen crasicolus*	Spain: Gerona(*n* = 4); Italy (*n* = 4); Morocco (*n* = 3)	WI	6.3
Medium fatty fishes				
Blue shark	*Prionace glauca*	Spain: Basque Country (*n* = 10), Cádiz (*n* = 1),	WI; SP	4.5
Swordfish	*Xiphias gladius*	Spain: Galicia (*n* =9), Huelva (*n* = 1)	WI; SP; SU	4.2
Red mullet	*Mulluss urmuletus*	Spain: Huelva (*n* = 8), Alicante (*n* = 3)	WI	3.8
Tuna	*Thunnus thynnus*	Spain: Canary Islands, (*n* = 7); Ghana (*n* = 3); Maldives (*n* = 1)	WI; SP; SU	3.3
White fishes				
Scad (horse mackerel)	*Trachurus trachurus*	Spain: Med coast (*n* = 10), Huelva (*n* = 1)	WI	2.0
Megrim	*Lepidor hombus boscii*	Spain: Atl. coast of Andalusia (*n* = 7), Alicante (*n* = 3)	WI; SP; AU	1.9
Hake	*Merluccius gayi*	Mauritania (*n* = 9); Spain: Basque Country (*n* = 1), Galicia (*n* = 1)	WI; SP; SU; AU	1.8
Perch*	*Lates niloticus*	Tanzania (*n* = 11)	WI; SP	1.6
European seabass	*Dicentrarchus labrax*	Spain: Murcia (*n* = 10), Granada (*n* = 1)	WI; SP	1.3
Common Sole	*Solea vulgaris*	Spain: Huelva (*n* = 9), Alicante (*n* = 2)	WI	1.3
Pangasius *	*Pangasius hypophthalmus*	Vietnam (*n* = 11)	WI; SP; AU	1.2
Codfish	*Gadus morhua*	Spain: Atlantic Northeast (*n* = 11)	**	1.0
European hake	*Merluccius merluccius*	Spain: Med. Coast (*n* = 10), Huelva (*n* = 1)	WI	0.8
Blue whiting	*Micromesistius poutassou*	Spain: Galicia (*n* = 9); Portugal (*n* = 2)	WI	0.7
Anglerfish	*Lophius piscatorius*	Mauritania (*n* = 11)	WI; AU	0.6
Mollusks and crustacean				
Mussel	*Mytilus edulis*	Spain: Galicia (*n* = 11)	WI	1.9
Shrimp	*Parapenaeus longirostris*	Spain: Huelva (*n* = 11)	WI	1.8
Clam	*Venus gallina*	Spain: Huelva (*n* = 2); Italy (*n* = 9)	WI	1.6
Squid	*Dosidicus gigas*	Argentina (*n* = 11)	WI; SP	1.5
Cuttlefish	*Sepia officinalis*	Spain: Huelva (*n* = 10), Alicante (*n* = 1)	WI; SP; SU	1.0

Fishes were classified depending on their lipid content: (1) fatty (6–25% fat), (2) medium fatty (2.5–6% fat) and (3) lean fish (<2.5% fat) [1]. * Indicates freshwater fish. ** Codfish was frozen, and the season of fishing is unknown. Fat composition data were collected from the Spanish food composition database (BEDCA) [17], except the blue shark, for which data were collected from the United States Department of Agriculture Food Composition Databases (USDA) [18]. Atl. coast: Atlantic coast; AU: autumn; Med. coast: Mediterranean coast; SP: spring; SU: summer; WI: winter.

**Table 2:** Percentage of fatty acids in fresh fatty and medium fatty fishes usually consumed in Spain.

	Fresh Fatty Fishes					Fresh Medium Fatty Fishes			
	Salmon (*n* = 11)	Mackerel (*n* = 11)	Sardine (*n* = 11)	Gilt-Head (*n* = 11)	Anchovy (*n* = 11)	Blue Shark (*n* = 11)	Swordfish (*n* = 10)	Red Mullet (*n* = 11)	Tuna (*n* = 8)
C12:0	0.02 ± 0.00	0.09 ± 0.01	n.d.	0.03 ± 0.01	0.12 ± 0.02	n.d.	0.02 ± 0.01	0.26 ± 0.04	n.d.
C13:0	0.01 ± 0.00	n.d.	n.d.	n.d.	n.d.	n.d.	0.01 ± 0.00	n.d.	n.d.
C14:0	1.9 ± 0.1	4.3 ± 0.7	9.4 ± 0.8	2.8 ± 0.3	6.0 ± 1.0	1.1 ± 0.1	1.4 ± 0.2	3.0 ± 0.2	2.9 ± 0.9
C14:1	0.01 ± 0.00	n.d.	n.d.	0.05 ± 0.01	n.d.	n.d.	n.d.	n.d.	n.d.
C15:0	0.1 ± 0.0	0.9 ± 0.1	n.d.	0.5 ± 0.1	1.1 ± 0.1	n.d.	0.2 ± 0.0	1.0 ± 0.1	n.d.
C16:0	9.0 ± 0.6	29.9 ± 1.2	39.0 ± 2.0	23.8 ± 1.8	37.7 ± 2.8	20.8 ± 0.6	17.6 ± 1.3	32.5 ± 0.9	32.6 ± 1.7
C16:1n-7	1.8 ± 0.1	3.3 ± 0.4	7.1 ± 0.4	6.1 ± 0.4	4.7 ± 0.7	3.3 ± 0.3	1.8 ± 0.2	6.6 ± 0.7	4.5 ± 1.0
C17:0	0.06 ± 0.01	0.9 ± 0.1	n.d.	n.d.	1.0 ± 0.1	n.d.	0.29 ± 0.05	1.2 ± 0.1	0.8 ± 0.1
C17:1n-7	0.05 ± 0.01	n.d.	n.d.	0.35 ± 0.10	n.d.	n.d.	0.30 ± 0.05	n.d.	n.d.
C16:2n-4	0.13 ± 0.02	n.d.	n.d.	n.d.	n.d.	n.d.	n.d.	n.d.	n.d.
C18:0	1.1 ± 0.1	8.0 ± 0.7	5.8 ± 0.3	4.3 ± 0.8	5.3 ± 0.1	13.2 ± 0.6	4.4 ± 0.7	7.1 ± 0.3	8.9 ± 0.6
C18:1n-9	60.1 ± 1.5	12.7 ± 2.8	10.9 ± 1.1	24.8 ± 3.2	8.3 ± 0.9	15.4 ± 0.8	54.0 ± 3.8	14.1± 1.4	20.6 ± 2.1
C18:1n-7	1.5 ± 0.1	3.1 ± 0.2	3.9 ± 0.2	2.7 ± 0.3	n.d.	5.7 ± 0.2	1.5 ± 0.1	n.d.	2.7 ± 0.2
*cis*C18:2n-6	10.5 ± 0.6	1.3 ± 0.1	1.9 ± 1.2	10.0 ± 2.1	1.2 ± 0.2	1.1 ± 0.2	n.d.	n.d.	0.8 ± 0.1
C20:0	n.d.	0.5 ± 0.1	n.d.	n.d.	0.5 ± 0.1	n.d.	n.d.	0.48 ± 0.04	0.33 ± 0.06
C18:3n-6	n.d.	n.d.	1.7 ± 0.2	0.15 ± 0.04	n.d.	n.d.	n.d.	n.d.	n.d.
C20:1n-9	3.2 ± 0.3	1.8 ± 0.8	n.d.	0.41 ± 0.12	n.d.	1.8 ± 0.2	3.8 ± 0.9	n.d.	1.3 ± 0.3
C18:3n-3	2.7 ± 0.2	0.6 ± 0.1	0.33 ± 0.05	0.8 ± 0.1	0.8 ± 0.2	n.d.	0.13 ± 0.02	n.d.	0.26 ± 0.04
C20:2n-6	0.9 ± 0.1	1.0 ± 0.1	0.8 ± 0.2	0.9 ± 0.1	n.d.	n.d.	0.20 ± 0.04	1.1 ± 0.1	0.7 ± 0.1
C22:0	n.d.	n.d.	n.d.	0.09 ± 0.02	0.23 ± 0.02	n.d.	0.08 ± 0.02	0.29 ± 0.02	n.d.
C20:3n-6	n.d.	n.d.	n.d.	0.15 ± 0.03	n.d.	n.d.	0.02 ± 0.00	n.d.	0.08 ± 0.01
C22:1n-9	0.21 ± 0.02	n.d.	n.d.	0.18 ± 0.04	n.d.	n.d.	0.5 ± 0.1	n.d.	0.27 ± 0.05
C20:3n-3	0.21 ± 0.05	n.d.	n.d.	0.05 ± 0.01	n.d.	n.d.	0.06 ± 0.01	n.d.	0.19 ± 0.05
C20:4n-6	0.18 ± 0.05	1.4 ± 0.2	0.8 ± 0.3	2.7 ± 1.0	1.2 ± 0.2	4.5 ± 0.5	0.6 ± 0.2	3.2 ± 0.2	1.8 ± 0.4
C22:2n-6	0.45 ± 0.04	n.d.	n.d.	0.41 ± 0.07	n.d.	0.35 ± 0.06	n.d.	n.d.	0.40 ± 0.07
C20:5n-3	1.9 ± 0.2	5.9 ± 0.7	7.2 ± 1.3	6.5 ± 0.8	7.7 ± 0.9	5.1 ± 0.3	0.9 ± 0.1	10.2 ± 0.7	3.7 ± 0.7
C24:1n-9	0.26 ± 0.05	1.2 ± 0.1	n.d.	0.38 ± 0.05	0.8 ± 0.1	1.1 ± 0.1	0.8 ± 0.3	0.49 ± 0.04	1.2 ± 0.2
C22:5n-3	0.8 ± 0.1	1.1 ± 0.1	1.6 ± 0.3	2.8 ± 0.4	0.6 ± 0.1	5.4 ± 0.3	1.0 ± 0.2	2.4 ± 0.2	1.0 ± 0.1
C22:6n-3	3.0 ± 0.4	22.0 ± 2.6	9.6 ± 1.8	9.1 ± 1.2	22.8 ± 3.8	21.3 ± 0.7	9.2 ± 2.0	16.0 ± 1.8	15.0 ± 2.9
SFA	12.1 ± 0.8	44.6 ± 1.9	54.2 ± 2.8	31.5 ± 2.4	51.8 ± 3.4	35.1 ± 0.8	25.0 ± 1.6	45.8 ± 1.1	45.5 ± 1.8
MUFA	67.1 ± 1.4	22.1 ± 3.0	22.1 ± 1.1	35.0 ± 2.8	13.8 ± 1.3	27.2 ± 1.1	62.8 ± 3.1	21.2 ± 1.9	30.5 ± 2.3
n-6 LC-PUFA	12.0 ± 0.5	3.7 ± 0.3	3.5 ± 1.3	13.9 ± 1.3	2.4 ± 0.9	5.9 ± 0.5	0.81 ± 0.21	4.3 ± 0.3	3.8 ± 0.5
n-3 LC-PUFA	8.7 ± 0.7	29.7 ± 3.2	18.8 ± 4.5	19.24 ± 2.2	31.9 ± 4.3	31.7 ± 0.6	11.37 ± 2.2	28.6 ± 2.1	20.2 ± 3.1
n-3/n-6 PUFA	0.72 ± 0.06	8.0 ± 0.6	4.3 ± 3.8	1.6 ± 0.4	13.7 ± 1.8	5.7 ± 0.5	17.4 ± 2.4	6.7 ± 0.5	5.4 ± 0.5
EPA/DHA	0.68 ± 0.05	0.28 ± 0.02	0.82 ± 0.10	0.80 ± 0.13	0.05 ± 0.01	0.24 ± 0.02	0.10 ± 0.03	0.69 ± 0.07	0.40 ± 0.19

Data are presented as mean percentages ± SEM. Fishes were classified depending on their lipid content: (1) fatty (6–25% fat), (2) medium fatty (2.5–6% fat) and (3) lean fish (<2.5% fat) [1]. DHA, docosahexaenoic acid; EPA, eicosapentaenoic acid; MUFA, monounsaturated fatty acid; n-3 LC-PUFA, n-3 long-chain polyunsaturated fatty acid; n-6 LC-PUFA, n-6 long-chain polyunsaturated fatty acid; n.d., non-detectable; SFA, saturated fatty acids.

**Table 3:** Percentage of fatty acids in fresh white fishes usually consumed in Spain.

	Scad (*n* = 11)	Megrim (*n* = 11)	Hake (*n* = 11)	Perch (*n* = 11)	European Seabass (*n* = 11)	Common Sole (*n* = 11)	Pangasius (*n* = 11)	Codfish (*n* = 11)	European Hake (*n* = 11)	Blue Whiting (*n* = 11)	Anglerfish (*n* = 11)
C12:0	0.16 ± 0.03	0.11 ± 0.01	0.03 ± 0.01	n.d.	0.02 ± 0.00	0.17 ± 0.03	0.17 ± 0.10	n.d.	n.d.	n.d.	n.d.
C14:0	4.6 ± 0.5	4.1 ± 0.6	1.6 ± 0.2	2.5 ± 0.2	2.3 ± 0.2	3.0 ± 0.4	1.8 ±0.3	1.7 ± 0.2	1.7 ± 0.1	0.9 ± 0.1	0.9 ± 0.1
C14:1	n.d.	n.d.	n.d.	n.d.	0.04 ± 0.00	n.d.	n.d.	n.d.	n.d.	n.d.	n.d.
C15:0	0.69 ± 0.07	0.80 ± 0.02	0.26 ± 0.02	0.46 ± 0.03	0.18 ± 0.02	0.9 ± 0.1	0.12 ± 0.03	0.37 ± 0.03	0.7 ± 0.1	0.35 ± 0.03	0.39 ± 0.03
C16:0	29.8 ± 1.6	26.6 ± 0.9	24.5 ± 1.5	30.6 ± 1.5	22.1 ± 1.1	28.1 ± 1.2	23.8 ± 0.4	23.3 ± 0.5	23.8 ± 0.7	25.7 ± 0.8	22.9 ± 1.5
C16:1n-7	4.7 ± 0.4	5.8 ± 0.5	4.5 ± 0.5	8.6 ± 1.0	4.3 ± 0.4	4.3 ± 0.4	0.5 ± 0.1	2.2 ± 0.2	2.2 ± 0.2	1.2 ± 0.1	4.5 ± 0.4
C17:0	0.8 ± 0.1	n.d.	0.28 ± 0.03	0.9 ± 0.0	0.17 ± 0.02	1.1 ± 0.1	0.09 ± 0.02	n.d.	0.7 ± 0.1	0.43 ± 0.04	n.d.
C17:1n-7	0.42 ± 0.04	n.d.	n.d.	0.43 ± 0.05	0.12 ± 0.02	0.9 ± 0.1	n.d.	n.d.	0.52 ± 0.03	0.7 ± 0.1	0.9 ± 0.1
C18:0	7.5 ± 0.5	5.4 ± 0.5	3.7 ± 0.4	8.9 ± 0.6	2.0 ± 0.2	9.7 ± 0.7	23.8 ± 0.3	3.2 ±0.2	6.6 ± 0.2	5.7 ± 0.2	7.1 ± 0.4
C18:1n-9	19.7 ± 2.4	11.9 ± 0.7	30.1 ± 4.1	14.6 ± 0.9	34.1 ± 2.1	8.0 ± 0.7	37.8 ± 2.5	7.6 ±0,3	10.1 ± 0.8	13.7 ± 1.0	13.3 ± 1.0
C18:1n-7	2.9 ± 0.2	2.9 ± 0.1	2.9 ± 0.1	2.9 ± 0.3	1.7 ± 0.1	4.3 ± 0.3	0.6 ± 0.1	3.6 ± 0,2	3.7 ± 0.8	3.1 ± 0.2	3.7 ± 0.3
*cis*C18:2n-6	1.2 ± 0.2	1.0 ± 0.1	0.9 ± 0.1	1.2 ± 0.1	14.0 ± 0.9	1.1 ± 0.1	6.4 ± 0.7	0.98 ± 0.05	1.0 ± 0.0	1.5 ± 0.1	1.3 ± 0.2
C20:0	0.5 ± 0.1	0.21 ± 0.04	0.23 ± 0.03	0.24 ± 0.02	n.d.	0.27 ± 0.04	0.11 ± 0.02	n.d.	0.21 ± 0.02	n.d.	n.d.
C18:3n-6	n.d.	n.d.	n.d.	n.d.	0.12 ± 0.01	n.d.	0.14 ± 0.03	n.d.	n.d.	n.d.	n.d.
C20:1n-9	1.6 ± 0.3	0.9 ± 0.1	4.4 ± 0.8	0.28 ± 0.02	1.1 ± 0.2	0.36 ± 0.04	0.5 ± 0.1	2.1 ± 0.3	0.6 ± 0.1	n.d.	0.8 ± 0.1
C18:3n-3	0.4 ± 0.1	0.22 ± 0.05	0.48 ± 0.17	1.2 ± 0.1	1.0 ± 0.1	0.23 ± 0.04	0.23 ± 0.03	n.d.	0.40 ± 0.03	n.d.	n.d.
C20:2n-6	n.d.	0.6 ± 0.1	0.6 ± 0.1	0.34 ± 0.02	1.0 ± 0.1	0.7 ± 0.1	0.37 ± 0.08	0.74 ± 0.06	0.7 ± 0.1	0.7 ± 0.1	0.22 ± 0.05
C22:0	n.d.	0.14 ± 0.01	n.d.	0.15 ± 0.02	0.03 ± 0.00	n.d.	0.05 ± 0.01	n.d.	n.d.	n.d.	n.d.
C20:3n-6	n.d.	0.05 ± 0.01	n.d.	0.20 ± 0.02	0.05 ± 0.01	n.d.	0.7 ± 0.2	n.d.	n.d.	n.d.	n.d.
C22:1n-9	n.d.	0.10 ± 0.01	0.7 ± 0.2	0.10 ± 0.02	0.10 ± 0.02	0.23 ± 0.03	0.06 ± 0.04	0.29 ± 0.04	n.d.	n.d.	0.18 ± 0.03
C20:3n-3	n.d.	n.d.	n.d.	0.25 ± 0.02	0.06 ± 0.01	n.d.	n.d.	n.d.	n.d.	n.d.	n.d.
C23:0	n.d.	0.18 ± 0.02	n.d.	n.d.	n.d.	n.d.	0.04 ± 0.02	n.d.	n.d.	n.d.	n.d.
C20:4n-6	1.0 ± 0.2	2.3 ± 0.2	1.3 ± 0.3	3.8 ± 0.8	0.32 ± 0.05	4.4 ± 0.2	1.2 ± 0.4	2.1 ± 0.2	2.3 ± 0.1	2.3 ± 0.2	6.5 ± 0.3
C22:2n-6	n.d.	n.d.	0.25 ± 0.05	n.d.	0.27 ± 0.03	0.24 ± 0.02	n.d.	n.d.	n.d.	n.d.	n.d.
C20:5n-3	5.1 ± 0.8	8.1 ± 1.0	4.9 ± 0.7	2.9 ± 0.2	6.5 ± 1.1	6.5 ± 0.5	0.20 ± 0.06	19.6 ± 0.8	7.5 ± 0.4	10.1 ± 0.5	6.2 ± 0.5
C24:1n-9	0.9 ± 0.1	0.9 ± 0.1	1.0 ± 0.1	0.41 ± 0.06	0.17 ± 0.03	n.d.	0.13 ± 0.03	n.d.	1.0 ± 0.1	n.d.	1.2 ± 0.1
C22:5n-3	1.6 ± 0.3	3.9 ± 0.2	1.2 ± 0.2	3.4 ± 0.3	0.82 ± 0.09	6.5 ± 0.3	0.26 ± 0.09	1.2 ± 0.1	1.1 ± 0.0	0.8 ± 0.1	2.1 ± 0.1
C22:6n-3	16.5 ± 3.0	23.9 ± 1.5	16.4 ± 3.2	15.5 ± 1.3	7.4 ± 1.1	19.1 ± 1.6	1.0 ± 0.3	31.0 ± 1,2	35.4 ± 1.0	33.1 ± 1.1	28.1 ± 2.0
SFA	44.0 ± 2.3	37.3 ± 1.2	30.5 ± 1.5	43.7 ± 1.0	26.9 ± 1.0	43.2 ± 2.0	50.0 ± 0.9	28.6 ± 0.7	33.5 ± 0.9	33.0 ± 0.9	31.3 ± 1.8
MUFA	30.2 ± 2.7	22.4 ± 1.2	43.5 ± 4.2	27.4 ± 1.6	41.6 ± 1.7	18.0 ± 1.2	40.0 ± 2.2	15.8 ± 0.7	18.1 ± 1.1	18.7 ± 1.2	24.5 ± 1.3
n-6 LC-PUFA	2.3 ± 0.3	3.9 ± 0.2	3.0 ± 0.4	5.6 ± 0.9	15.6 ± 0.9	6.5 ± 0.2	8.8 ± 1.3	3.8 ± 0.2	4.0 ± 0.1	4.5 ± 0.2	7.9 ± 0.3
n-3 LC-PUFA	23.6 ± 4.0	36.3 ± 1.8	22.9 ± 4.0	23.2 ± 1.5	14.8 ± 1.6	32.3 ± 2.0	1.7 ± 0.5	51.8 ± 1.2	44.4 ± 1.1	43.9 ± 1.2	36.3 ± 2.5
n-3/n-6 PUFA	10.0 ± 1.1	9.4 ± 0.5	7.4 ± 0.7	4.7 ± 0.4	1.0 ± 0.2	5.0 ± 0.3	0.16 ± 0.03	14.2 ± 0.9	11.1 ± 0.3	10.1 ± 0.4	4.5 ± 0.2
EPA/DHA	0.31 ± 0.03	0.35 ± 0.05	0.34 ± 0.03	0.19 ± 0.01	1.05 ± 0.24	0.36 ± 0.03	0.24 ± 0.05	0.64 ± 0.04	0.21 ± 0.01	0.31 ± 0.02	0.22 ± 0.01

Data are presented as mean percentages ± SEM. Fishes were classified depending on their lipid content: (1) fatty (6–25% fat), (2) medium fatty (2.5–6% fat) and (3) lean fish (<2.5% fat) [1]. DHA, docosahexaenoic acid; EPA, eicosapentaenoic acid; MUFA, monounsaturated fatty acid; n-3 LC-PUFA, n-3 long-chain polyunsaturated fatty acid; n-6 LC-PUFA, n-6 long-chain polyunsaturated fatty acid; n.d., non-detectable; SFA, saturated fatty acids.

**Table 4:** Percentages of fatty acids in fresh mollusks and crustaceans usually consumed in Spain.

	Shrimp (*n* = 11)	Mussel (*n* = 10)	Clam (*n* = 11)	Squid (*n* = 11)	Cuttlefish (*n* = 11)
C12:0	n.d.	n.d.	0.27 ± 0.09	n.d.	n.d.
C14:0	4.4 ± 1.0	2.5 ± 0.3	3.1 ± 0.3	1.7 ± 0.1	2.5 ± 0.2
C15:0	1.1 ± 0.1	1.0 ± 0.1	0.80 ± 0.04	0.43 ± 0.02	0.71 ± 0.03
C16:0	19.3 ± 0.4	31.1 ± 1.9	33.3 ± 0.8	30.6 ± 0.2	23.3 ± 0.4
C16:1n-7	3.6 ± 0.2	8.9 ± 1.0	5.1 ± 0.5	0.55 ± 0.05	1.4 ± 0.3
C17:0	1.4 ± 0.1	1.2 ± 0.1	2.0 ± 0.2	0.40 ± 0.02	1.4 ± 0.1
C18:0	6.9 ± 0.2	4.7 ± 0.3	8.3 ± 0.4	3.3 ± 0.4	9.04 ± 0.7
C18:1n-9	14.0 ± 0.8	1.6 ± 0.1	4.4 ± 0.2	2.5 ± 0.2	6.4 ± 1.3
C18:1n-7	4.4 ± 0.3	3.2 ± 0.1	2.8 ± 0.3	1.2 ± 0.0	2.20 ± 0.2
*cis*C18:2n-6	1.5 ± 0.1	2.4 ± 0.2	1.7 ± 0.2	0.35 ± 0.06	2.4 ± 0.5
C20:1n-9	0.8 ± 0.1	2.6 ± 0.2	n.d.	3.3 ± 0.1	2.8 ± 0.3
C18:3n-3	n.d.	0.75 ± 0.07	3.4 ± 0.2	n.d.	n.d.
C20:2n-6	1.5 ± 0.1	1.8 ± 0.2	n.d.	0.31 ± 0.02	0.96 ± 0.13
C22:1n-9	n.d.	n.d.	n.d.	0.93 ± 0.06	n.d.
C20:4n-6	5.1 ± 0.3	3.2 ± 0.4	4.1 ± 0.3	0.73 ± 0.10	4.2 ± 0.4
C20:5n-3	15.0 ± 0.5	18.8 ± 1.2	11.4 ± 0.5	12.4 ± 0.3	16.5 ± 0.7
C22:5n-3	1.5 ± 0.1	1.3 ± 0.1	2.6 ± 0.2	n.d.	1.5 ± 0.1
C22:6n-3	19.5 ± 0.8	14.9 ± 1.5	16.9 ± 1.1	41.5 ± 0.6	24.6 ± 1.4
SFA	33.2 ± 1.2	40.6 ± 2.1	47.8 ± 0.9	36.3 ± 0.3	36.9 ± 0.9
MUFA	22.9 ± 0.9	16.3 ± 1.0	12.2 ± 0.9	8.4 ± 0.3	12.8 ± 1.6
n-6 LC-PUFA	8.0 ± 0.4	7.4 ± 0.5	5.8 ± 0.3	1.3 ± 0.1	7.6 ± 0.6
n-3 LC-PUFA	36.0 ± 1.3	35.7 ± 2.5	34.2 ± 1.4	54.0 ± 0.6	42.6 ± 2.0
n-3/n-6 PUFA	4.6 ± 0.2	4.9 ± 0.2	6.1 ± 0.4	41.1 ± 2.7	6.2 ± 0.8
EPA/DHA	0.77 ± 0.02	1.3 ± 0.1	0.70 ± 0.05	0.30 ± 0.01	0.68 ± 0.04

Data are presented as mean percentages ± SEM. DHA, docosahexaenoic acid; EPA, eicosapentaenoic acid; MUFA, monounsaturated fatty acid; n-3 LC-PUFA, n-3 long-chain polyunsaturated fatty acid; n-6 LC-PUFA, n-6 long-chain polyunsaturated fatty acid; n.d., non-detectable; SFA, saturated fatty acids.

**Table 5:** Estimation of the amount of n-3 provided per gram of seafood, per serving (150 g) and per day after ingestion of one serving of fatty, medium fatty, and white fresh fishes, mollusks and crustaceans that are typically consumed in Spain.

	Fatty Acids per Serving (g fat/150 g)	Amount of n-3 LC-PUFA (mg) per Gram of Seafood	Amount of n-3 LC-PUFA (g) per Serving of Seafood (150 g)	mg/d of n-3 LC-PUFA Provided with the Ingestion of One Serving per Week
Fatty fishes				
Salmon	16.2	9 ± 1	1.4 ± 0.1	200 ± 17
Mackerel	16.1	32 ± 3	4.8 ± 0.5	681 ± 74
Sardine	10.1	13 ± 2	1.9 ± 0.3	271 ± 45
Gilt-head	9.7	12 ± 1	1.9 ± 0.2	267 ± 30
Anchovy	8.5	18 ± 2	2.7 ± 0.4	388 ± 52
Medium fatty fishes				
Blue shark	6.1	13 ± 0	1.9 ± 0.0	276 ± 5
Swordfish	5.7	4 ± 1	0.6 ± 0.1	92 ± 18
Red mullet	5.0	10 ± 1	1.4 ± 0.1	204 ± 15
Tuna	4.5	6 ± 1	0.9 ± 0.1	129 ± 20
White fishes		±	±	±
Scad	2.1	3 ± 1	0.5 ± 0.1	70 ± 12
Megrim	2.0	5 ± 0	0.7 ± 0.0	104 ± 5
Hake	1.9	3 ± 0	0.4 ± 0.1	62 ± 11
Perch *	1.7	3 ± 0	0.4 ± 0.0	56 ± 4
European seabass	1.4	1 ± 0	0.2 ± 0.0	31 ± 3
Common Sole	1.4	3 ± 0	0.4 ± 0.0	63 ± 4
Pangasius *	1.3	0 ± 0	0.0 ± 0.0	3 ± 1
Codfish	1.1	4 ± 0	0.5 ± 0.0	78 ± 2
European hake	0.9	3 ± 0	0.4 ± 0.0	56 ± 1
Blue whiting	0.7	2 ± 0	0.3 ± 0.0	46 ± 1
Anglerfish	0.6	2 ± 0	0.2 ± 0.0	33 ± 2
Fresh mollusks and crustacean				
Shrimp	1.9	5 ± 0	0.7 ± 0.0	98 ± 3
Mussel	2.0	5 ± 0	0.7 ± 0.1	102 ± 7
Clam	1.7	4 ± 0	0.6 ± 0.0	83 ± 3
Squid	1.5	6 ± 0	0.8 ± 0.0	119 ± 1
Cuttlefish	1.1	3 ± 0	0.4 ± 0.0	64 ± 3

Fishes were classified depending on their lipid content: 1) fatty (6–25% fat), 2) medium fatty (2.5–6% fat) and 3) lean fish (<2.5% fat) [1]. * Indicates freshwater fish. The calculation of the amount of fatty acid per serving is based on data collected from the Spanish food composition database (BEDCA) [17], except the blue shark, for which data are from the United States Department of Agriculture Food Composition Databases (USDA) [18], multiplied by a correction factor based on the average chain length of the fatty acids [21]. The suggested factor is 0.9 for fatty fish, and medium fatty fish have been considered as fatty fish for this factor. The suggested factor is 0.7 for lean fish, and mollusks and crustaceans have been considered as lean fish for this factor. To convert percentages of individual fatty acids to mg/g fish we multiplied the percentage of each individual fatty acid by the amount of total fatty acids calculated for the fish and divided by 100. Individual n-3 LC-PUFA were added to calculate total n-3 LC-PUFA (mg/g fish) included in Table 5. A serving of seafood is considered to weigh 150 g [22]. To calculate the amount of n-3 LC-PUFA (g) per serving of seafood (150 g) we multiplied the amount of n-3 LC-PUFA fatty acids per g fish by 150 g. Estimations of the amount of how much n-3 LC-PUFA would be provided per day after the ingestion of one serving per week of fishery products were calculated considering the amount of total n3- LC-PUFA provided by one serving divided per 7 days and expressed as mg/d. These data must only be taken as a guide or approximation.

## Data Availability

Not applicable.

## Supplementary Materials

The following are available online at https://www.mdpi.com/2072-6643/13/2/465/s1, Table S1: Retention times of analyzed fatty acids, Table S2: Amount of fatty acid (mg) per gram of fresh fatty and medium fatty fishes usually consumed in Spain, Table S3: Amount of fatty acid (mg) per gram of fresh white fishes usually consumed in Spain, Table S4: Amount of fatty acid (mg) per gram of fresh mollusks and crustaceans usually consumed in Spain.
