# Supplementary material for: Nutritional Importance of Selected Fresh Fishes, Shrimps and Mollusks to Meet Compliance with Nutritional Guidelines of n-3 LC-PUFA Intake in Spain
Source: Nutrients. 2021 Jan 30;13(2):465. doi: 10.3390/nu13020465 (PMC7911951; doi:10.3390/nu13020465)
Supplement: Supplementary file 1 [file nutrients-13-00465-s001.pdf]

Supplemental Table S1 Retention times of analyzed fatty acids

	Name	Retention time (min)
C12:0	Lauric acid	16.02
C13:0	Tridecanoic acid	17.54
C14:0	Myristic acid	19.16
C14:1	Myristoleic acid	20.64
C15:0	Pentadecanoic acid	20.82
C16:0	Palmitic acid	22.46
C16:1n-7	Palmitoleic acid	23.70
C17:0	Heptadecanoic acid	24.07
C17:1n-7	cis 11-heptadecenoic acid	25.28
C16:2n-4	cis 9,12-hexadienoic acid	25.40
C18:0	Stearic acid	25.64
C18:1n-9	Oleic acid	26.67
C18:1n-7	Elaidic acid	26.85
*cis*C18:2n-6	Linoleic acid	28.17
C20:0	Araquidic acid	28.58
C18:3n-6	Gamma-linolenic acid	29.26
C20:1n-9	Cis 11-eicoenoic acid	29.53
C18:3n-3	Linolenic acid	29.84
C20:2n-6	cis 11,14-eicosadienoic acid	30.94
C22:0	Behenic acid	31.29
C20:3n-6	cis 8,11,14-eicosatrienoic acid	31.97
C22:1n-9	Erucic acid	32.19
C20:3n-3	cis 11,14,17-eicosatrienoic acid	32.48
C20:4n-6	Araquidonic acid	32.75
C22:2n-6	cis 13,16-docosadienoic acid	33.51
C20:5n-3	cis 5,8,11,14,17-eicosapentadienoic acid (EPA)	34.40
C24:1n-9	Nervonic acid	34.74
C22:5n-3	cis 7,10,13,16,19-docosahexadienoic acid (DPA)	37.27
C22:6n-3	cis 4,7,10,13,16,19-docosahexadienoic acid (DHA)	38.09

Supplemental Table S2 Amount of fatty acid (mg) per gram of fresh fatty and medium fatty fishes usually consumed in Spain.

	Fresh fatty fishes					Fresh medium fatty fishes			
	**Salmon (*n* = 11)**	**Mackerel (*n* = 11)**	**Sardine (*n* = 11)**	**Gilt-head (*n* = 11)**	**Anchovy (*n* = 11)**	**Blue shark (*n* = 11)**	**Swordfish (*n* = 11)**	**Red mullet (*n* = 11)**	**Tuna (*n* = 8)**
C14:0	2.0 ± 0	5 ± 1	6 ± 1	2 ± 0	3 ± 1	<1	1 ± 0	1 ± 0	1 ± 0
C15:0	<1	1 ± 0	<1	<1	1 ± 0	<1	<1	<1	<1
C16:0	10 ± 1	32 ± 1	26 ± 1	15 ± 1	21 ± 2	8 ± 0	7 ± 0	11 ± 0	10 ± 1
C16:1n-7	2 ± 0	4 ± 0	5 ±0	4 ± 0	3 ± 0	1 ± 0	1 ± 0	2 ± 0	1 ± 0
C17:0	<1	1 ± 0	<1	<1	1 ± 0	<1	<1	<1	<1
C18:0	1 ± 0	9 ± 1	4 ±0	3 ± 1	3 ± 0	5 ± 0	2 ± 0	2 ± 0	3 ± 0
C18:1n-9	65 ± 2	14 ± 3	7 ±1	16 ± 2	5 ± 0	6 ± 0	20 ± 1	5 ± 0	6 ± 1
C18:1n-7	2 ± 0	3 ± 0	3 ± 0	2 ± 0	<1	2 ± 0	1 ± 0	<1	1 ± 0
*cis*C18:2n-6	11 ± 1	1 ± 0	1 ± 1	6 ± 1	1 ± 0	<1	<1	<1	<1
C20:0	<1	1 ± 0	<1	<1	<1	<1	<1	<1	<1
C20:1n-9	4 ± 0	2 ± 1	<1	<1	<1	1 ± 0	1 ± 0	<1	<1
C18:3n-3	3 ± 0	1 ± 0	<1	1 ± 0	<1	<1	<1	<1	<1
C20:2n-6	1 ± 0	1 ± 0	1 ± 0	1 ± 0	<1	<1	<1	<1	<1
C20:4n-6	<1	1 ± 0	1 ±0	2 ± 1	1 ± 0	2 ± 0	<1	1 ± 0	1 ± 0
C20:5n-3	2 ± 0	6 ± 1	5 ± 1	4 ± 1	4 ± 1	2 ± 0	<1	3 ± 0	1 ± 0
C24:1n-9	<1	1 ± 0	<1	<1	<1	<1	<1	<1	<1
C22:5n-3	1 ± 0	1 ± 0	1 ± 0	2 ± 0	<1	2 ± 0	<1	1 ± 0	<1
C22:6n-3	3 ± 0	24 ± 3	6 ± 1	6 ± 1	13 ± 2	9 ± 0	3 ± 0	5 ± 1	4 ± 1
SFA	13 ± 1	48 ± 2	37 ± 2	20 2±	29 ± 2	14 ± 0	9 ± 1	15 ± 0	14 ± 1
MUFA	72 ± 2	24 ± 3	15 ± 1	23 ± 2	8 ± 1	11 ± 0	24 ± 1	7 ± 1	9 ± 1
n-6 LC-PUFA	13 ± 1	4 ± 0	4 ± 1	9 ± 1	1 ± 0	2 ± 0	0 ± 0	1 ± 0	1 ± 0
n-3 LC-PUFA	9 ± 1	32 ± 3	13 ± 2	12 ± 1	18 ± 2	13 ± 0	4 ± 1	10 ± 1	6 ± 1
n-3/n-6 PUFA	7 ± 1	80 ± 6	43 ± 8	15 ± 3	137 ± 18	57 ± 5	175 ± 24	67 ± 4	54 ± 5

EPA/DHA	0.7 ± 0.0	0.3 ± 0.1	8.2 ± 1.0	8.0 ± 1.3	0.5 ± 0.1	2.4 ± 0.2	0.1 ± 0.1	6.9 ± 0.7	4.0 ± 1.9

Data are presented in mg/g of fish, as the mean ± SEM. To calculate mg/g fatty and medium fatty fish, we multiply the percentage of each individual fatty acid by the amount of total fat described in BEDCA [17] and USDA (for the blue shark) [18] and by the suggested correction factor: 0.9 [21]. Then data were expressed as mg/g fish. DHA, docosahexaenoic acid; EPA, eicosapentaenoic acid; MUFA, monounsaturated fatty acid; n-3 LC-PUFA, n-3 long chain polyunsaturated fatty acid; n-6 LC-PUFA, n-6 long chain polyunsaturated fatty acid; SFA, saturated fatty acids.

Supplemental Table S3 Amount of fatty acid (mg) per gram of fresh white fishes usually consumed in Spain.

	**Scad (*n* = 11)**	**Megrim (*n* = 11)**	**Hake (*n* = 11)**	**Perch (*n* = 11)**	**European seabass (*n* = 11)**	**Common Sole (*n* = 11)**	**Pangasius (*n* = 11)**	**Codfish (*n* = 11)**	**European hake (*n* = 11)**	**Blue whiting (*n* = 11)**	**Anglerfish (*n* = 11)**
C14:0	1 ± 0	1 ± 0	<1	<1	<1	<1	<1	<1	<1	<1	<1
C16:0	4 ± 0	4 ± 0	3 ± 0	3 ± 0	2 ± 0	3 ± 0	2 ± 0	2 ± 0	1 ± 0	1 ± 0	1 ± 0
C16:1n-7	1 ± 0	1 ± 0	1 ± 0	1 ± 0	<1	<1	<1	<1	<1	<1	<1
C18:0	1 ± 0	1 ± 0	<1	1 ± 0	<1	1 ± 0	2 ± 0	<1	<1	<1	<1
C18:1n-9	3 ± 0	2 ± 0	4 ± 1	2 ± 0	3 ± 0	1 ± 0	3 ± 0	1 ± 0	1 ± 0	1 ± 0	1 ± 0
*cis*C18:2n-6	<1	<1	<1	<1	1 ± 0	<1	1 ± 0	<1	<1	<1	<1
C20:1n-9	<1	<1	1 ± 0	<1	<1	<1	<1	<1	<1	<1	<1
C20:5n-3	1 ± 0	1 ± 0	1 ± 0	<1	1 ± 0	1 ± 0	<1	1 ± 0	<1	<1	<1
C22:6n-3	2 ± 0	3 ± 0	2 ± 0	2 ± 0	1 ± 0	2 ± 0	<1	2 ± 0	2 ± 0	2 ± 0	1 ± 0
SFA	6 ± 0	5 ± 0	4 ± 0	5 ± 0	2 ± 0	4 ± 0	4 ± 0	2 ± 0	2 ± 0	2 ± 0	1 ± 0
MUFA	4 ± 0	3 ± 0	5 ± 1	3 ± 0	4 ± 0	2 ± 0	3 ± 0	1 ± 0	1 ± 0	1 ± 0	1 ± 0
n-6 LC-PUFA	<1	1 ± 0	<1	1 ± 0	1 ± 0	1 ± 0	1 ± 0	<1	<1	0 ± 0	<1
n-3 LC-PUFA	3 ± 1	5 ± 0	3 ± 0	3 ± 0	1 ± 0	3 ± 0	<1	4 ± 0	3 ± 0	2 ± 0	2 ± 0
n-3/n-6 PUFA	100 ± 11	94 ± 5	74 ± 7	47 ± 4	11 ± 2	49 ± 3	2 ± 0	142 ± 9	111 ± 3	101 ± 4	45 ± 2
EPA/DHA	3.1 ± 0.3	3.5 ± 0.5	3.4 ± 0.3	1.9 ± 0.1	10.5 ± 2.4	3.6 ± 0.3	2.4 ± 0.5	6.4 ± 0.4	2.1 ± 0.1	3.1 ± 0.2	2.2 ± 0.1

Data are presented in mg/g of fish, as the mean ± SEM. To calculate mg/g lean fish, we multiplied the percentage of each individual fatty acid by the amount of total fat described in BEDCA [17] and by the suggested correction factor: 0.7 [21]. Then data were expressed as mg/g fish. DHA, docosahexaenoic acid; EPA, eicosapentaenoic acid; MUFA, monounsaturated fatty acid; n-3 LC-PUFA, n-3 long chain polyunsaturated fatty acid; n-6 LC-PUFA, n-6 long chain polyunsaturated fatty acid; SFA, saturated fatty acids.

Supplemental Table S4 Amount of fatty acid (mg) per gram of fresh mollusks and crustaceans usually consumed in Spain.

	**Shrimp (*n* = 11)**	**Mussel (*n* = 10)**	**Clam (*n* = 11)**	**Squid (*n* = 11)**	**Cuttlefish (*n* = 11)**
C14:0	1 ± 0	<1	<1	<1	<1
C16:0	2 ± 0	4 ± 0	4 ± 0	3 ± 0	2 ± 0
C16:1n-7	<1	1 ± 0	1 ± 0	<1	<1
C18:0	1 ± 0	1 ± 0	1 ± 0	<1	1 ± 0
C18:1n-9	2 ± 0	<1	<1	<1	<1
C18:1n-7	1 ± 0	<1	<1	<1	<1
C20:4n-6	1 ± 0	<1	<1	<1	<1
C20:5n-3	2 ± 0	2 ± 0	1 ± 0	1 ± 0	1 ± 0
C22:6n-3	2 ± 0	2 ± 0	2 ± 0	4 ± 0	2 ± 0
SFA	4 ± 0	5 ± 0	5 ± 0	4 ± 0	2 ± 0
MUFA	3 ± 0	2 ± 0	1 ± 0	1 ± 0	1 ± 0
n-6 LC-PUFA	1 ± 0	1 ± 0	1 ± 0	<1	1 ± 0
n-3 LC-PUFA	5 ± 0	5 ± 0	4 ± 0	6 ± 0	3 ± 0
n-3/n-6 PUFA	46 ± 2	49 ± 2	61 ± 4	411 ± 27	62 ± 8
EPA/DHA	7.7 ± 0.2	13.5 ±1.1	7.0 ± 0.5	3.0 ± 0.1	6.8 ± 0.4

Data are presented in mg/g of fish, as the mean ± SEM. To calculate mg/g mollusk and crustaceans, we multiply the percentage of each individual fatty acid by the amount of total fat described in BEDCA [17] and by the suggested correction factor: 0.7 [21]. Then data were expressed as mg/g mollusk and crustaceans. DHA, docosahexaenoic acid; EPA, eicosapentaenoic acid; MUFA, monounsaturated fatty acid; n-3 LC-PUFA, n-3 long chain polyunsaturated fatty acid; n-6 LC-PUFA, n-6 long chain polyunsaturated fatty acid; SFA, saturated fatty acids.
